# Role of emotion regulation capacities in affective state among Chinese high school students in the post-pandemic era of COVID-19

**DOI:** 10.3389/fpsyg.2022.1015433

**Published:** 2022-12-08

**Authors:** Suyan Wang, Yuying Chu, Hongliang Dai

**Affiliations:** ^1^Centre for Mental Health Guidance, Jinzhou Medical University, Jinzhou, China; ^2^School of Nursing, Jinzhou Medical University, Jinzhou, Liaoning, China

**Keywords:** adolescent, COVID-19, mental health, emotion regulation, affective state

## Abstract

**Objective:**

Psychological wellbeing and emotion regulation skills of vulnerable adolescents have been severely threatened by the long-term impact of the ongoing COVID-19 pandemic. The aim of this study was to seek out the potentially effective emotion regulation strategies to minimize the mental health risk of adolescents during the COVID-19 post-pandemic era.

**Methods:**

A total of 436 high school students aged 16.07 ± 1.08 years were included in this cross-sectional study to complete questionnaires to self-report socio-demographic information, positive and negative affect state, and emotional regulation abilities. Student's *t*-test and one-way analysis of variance (ANOVA) were used for intergroup comparisons among socio-demographic variables. Pearson's correlation analysis was used for evaluating the association between each emotion regulation strategy and positivity or negativity. Multiple stepwise linear regression analysis was used for the determination of the predictors for adolescents' positivity and negativity.

**Results:**

Adolescents' affect was influenced by multiple emotion regulation strategies, including cognitive reappraisal, acceptance and engagement, difficulty in awareness, acceptance, and modification of emotions after adjusting for a range of socio-demographic variables.

**Conclusion:**

Overall, our findings highlight the importance of emotional regulation strategies in the modulation of the mental health of the vulnerable youth population in China during the COVID-19 crisis. In view of the continuous, multifaceted influence on adolescents' mental health of the ongoing pandemic, more effort should be made to leverage emotion regulation strategies to benefit their coping abilities.

## Introduction

Since being announced as a pandemic by the World Health Organization (WHO) in March 2020, coronavirus disease 2019 (COVID-19) has swept the globe for more than 2 years. The pandemic and related containment measures such as nationwide or regional lockdown have triggered a plethora of the mental health problems such as anxiety, depression, and post-traumatic stress disorder (PTSD) among affected individuals, including adolescents (Zhang et al., 2020; Murata et al., 2021; Wu et al., 2021). Adolescence is especially sensitive to stress exposures and vulnerable to the negative influence of the COVID-19 pandemic (Berger et al., 2021; Cao et al., 2022). Extant studies have confirmed the negative influence of the pandemic on mental health of adolescents (Wu et al., 2021; Okajima et al., 2022). Notably, the influence of the pandemic on mental health not only works during the pandemic but also has long-term mental health consequences after the pandemic and lockdown (Awan et al., 2021; Cao et al., 2022). As such, it is critically important to address the mental health problems during the post-pandemic era.

Mental health research nowadays has gradually shifted away from focusing on clinically relevant mental illness toward emphasizing human wellbeing and flourishing in the perspective of positive psychology (Keyes, 2007; Choon et al., 2022; Paz et al., 2022). According to the proposition by Keyes, mental health should be considered more than the absence of mental illness and a continuum from flourishing mental health to languishing mental health (Keyes, 2007). Flourishing individuals are characterized as being psychologically and socially functional and enjoy high levels of meaning in life, happiness, positive relationships, self-acceptance, and subjective wellbeing. Languishing ones, however, claim to lead an empty and stagnant life. It is worth noting that the negative influence of languishing on emotional health is comparable to that of major depression (Keyes, 2007). In this sense, mental disorders can be deemed as emotional illness (Keyes, 2002). Therefore, individuals' mental health level is closely related to their affective state. Studies have shown that the balance of positive to negative affect is closely related to wellbeing and mental health status, with a higher positivity ratio predicting better mental health (Diehl et al., 2011). Specifically, studies have suggested the notion of a specific positivity ratio distinguishing different mental health statuses. For example, a critical ratio of 2.9 is able to well discriminate young adults and college students with flourishing mental health from those not flourishing (Fredrickson and Losada, 2005; Diehl et al., 2011). A wide spectrum of empirical evidence also asserts that positive feelings result in salubrious mental health outcomes and promote the development of varied beneficial psychological characteristics, such as resilience, happiness, and psychological growth (Fredrickson and Joiner, 2002; Fredrickson et al., 2003).

Emotional regulation refers to the abilities or coping strategies to effectively address psychological distress based on cognitive and behavioral tactics (Tao et al., 2022). Individuals' affective states are remarkably modulated by emotional regulation strategies. In a recent study, Wante et al. (2018) examined and compared the efficiency of diverse emotion regulation strategies in early adolescents and found that distraction significantly increased positive affect and decreased negative affect, following after watching an emotion-eliciting short film. In contrast, a long-term and recurrent rumination is assumed to cause an increase in negative affect in adolescents (Park et al., 2004). Emotional regulation insufficiency or inappropriateness was also found to be associated with a plethora of mental disorders, such as depression, anxiety, and suicidal ideation (Marganska et al., 2013; Brausch and Woods, 2019; Tao et al., 2022). Emotion regulation capacities are also critically important for the improvement of adolescents' emotional experience and their accommodation with socio-emotional stressors (McLaughlin et al., 2011; Ma and Fang, 2019). To sum up, the present study acknowledges mental health as a continuum ranging from psychologically or emotionally flourishing to languishing, and thus, it is of significance to understand the potential influence of diverse emotional regulation strategies on affective state among adolescents during this special post-pandemic era of COVID-19.

To date, multiple emotion regulation models have been proposed in the extant literature, such as emotional regulation process (Gross and John, 2003), difficulties in emotion regulation (Gratz and Roemer, 2004), adaptive coping with emotion (ACE) model, and affect regulation training (ART). According to Gross (1998), emotion regulation can be defined as the process through which individuals modulate what, when, and how they experience and manifest emotions. The whole process targets five major steps: situation selection, situation modification, deployment of attention, cognitive change, and response modification. The emotion regulation process theory classified these five key points into antecedent-focused and response-focused tactics when confronted with unfavorable situations. Antecedent-focused strategies occur at the earlier stage of emotion-generating process and emphasize the modification of the input of emotion prior to actual emotion arousal, whereas response-focused strategies occur relatively later and emphasize the alteration of emotional response after emotions have been fully elicited. These two emotion regulation strategies can be quantitatively measured by the Emotion Regulation Questionnaire (ERQ), which contains two dimensions of cognitive reappraisal and expressive suppression. The former is of typical antecedent-focused strategy and the later response-focused strategy (Gross and John, 2003). Cognitive reappraisal has been widely deemed as adaptive emotion regulation skills and is of effective strategies to maintain mental wellbeing (Gross and John, 2003; Tao et al., 2022). The effectiveness of expressive suppression in mood recovery and mental health, however, is still controversial. Although expressive suppression is frequently found to be maladaptive and results in overwhelming emotions and further psychological distress (Gross and John, 2003; El Archi et al., 2022), much evidence confirms that this strategy seems less detrimental for individual's mental wellbeing, especially in the Asian cultural background (Su et al., 2015).

Difficulties in emotion regulation is an alternative emotion regulation theory which assumes that the adaptive emotion regulation process is composed of a range of aspects, including awareness, clarity, acceptance, modulation of emotion arousal, control of impulse, and goal-directed engagement even in the context of suppression (Gratz and Roemer, 2004). Dysfunction in these aspects would result in emotional problems, which can be measured using the Difficulties in Emotion Regulation Scale (DERS). This scale performed well in evaluating adolescents' emotion regulation and their internalizing/externalizing psychopathology (Neumann et al., 2010). A recent study revealed that DERS, especially acceptance and strategies, mediated the relationship between mindfulness and psychological distress among Chinese adolescents (Ma and Fang, 2019).

ACE and ART models were established to explain the mechanisms of emotional dysregulation and help develop effective interventions to improve emotion regulation. This model conceptualizes emotion regulation as a range of skills, including awareness, tolerance, acceptance, modification, readiness to confront, self-support, clarification, and understanding. The Emotion Regulation Skill Questionnaire (ERSQ) was developed to assess these emotion regulation skills above the ACE and ART model (eight skills) and the capability to interpret emotion-related bodily sensation (one skill). The original nine-factor ERSQ has shown satisfactory validity and reliability in Germans (Berking and Znoj, 2008). However, a recent psychometric measurement regarding this instrument in Japan showed that this measure was of a two-factor structure (Fujisato et al., 2017). The first factor included tolerance, modification, readiness to confront, and acceptance and accordingly named *Acceptance and Engagement*. This skill refers to willingness to accept and resign oneself to certain emotions. The second included sensation, awareness, understanding, clarification, and compassionate self-support and thus were named *Awareness and Understanding*. This skill refers to consciously and self-compassionately perceive and understand certain emotional arousal (Fujisato et al., 2017).

The emotional regulation strategies derived from these well-documented theoretical models above provide differential perspectives in addressing human emotion and affection, and in real life, these strategies are supposed to be comprehensively applied when individuals are experiencing negative emotions evoked by an adversity, as each strategy seems not to be always effective or maladaptive in regulating emotional response. Taking the two strategies, cognitive appraisal and expressive suppression, in emotional regulation process model as an example, cognitive reappraisal involves the reassessment and altered interpretation of emotion-eliciting conditions and is frequently found to be protective against emotional disorders and negative affect. In spite of that, a few studies also show that some individuals experience reappraisal failure. They just cannot effectively deploy this strategy to effectively buffer emotional intensity when facing certain emotion-eliciting situations (Gardener et al., 2013; Sarlo et al., 2013; Cao et al., 2020). Similarly, although expressive suppression was documented to be associated with low self-esteem, low adaptability, and high level of social anxiety on many occasions (Bebko et al., 2014), this strategy seems conducive in other situations, for example, when perceiving anxiety during the job interview, or when being praised due to doing better than peers (Heiy and Cheavens, 2014). In a word, the exact role of certain emotional regulation strategy in shaping affective state might be distinct under different social and cultural surroundings.

The post-pandemic era has exerted an immense and long-lasting negative impacts on adolescents' mental health due to the unpredictable and sporadic outbreak of COVID-19. Individuals are unable to live a normal life in this special period and would frequently experience intolerance of uncertainty and impermanence (Korte et al., 2022). In addition, people have to comply with management and control measures, like traveling limits, social distancing, and sudden quarantine, and have to experience dramatic changes in the living environment. All these are expected to aggravate psychological and emotional disorders among the adolescent population (Wang S. et al., 2022). To help contain the overall deterioration of adolescents' mental health in this special and unprecedented period, an academic and practical concern regarding the effectiveness of commonly used emotional regulation strategies should have been addressed *via* empirical research, that is, which emotion regulation strategies are truly effective for maintaining Chinese adolescents' favorable affect state during the post-pandemic age of COVID-19. The extant findings drive the hypothesis that certain emotion regulation strategies are potentially implicated in the modulation of affect state among Chinese high school students in the post-pandemic era of COVID-19. In the current study, we aimed to assess the emotion regulation capacities from multiple perspectives by using distinct measures and to analyze whether the emotion regulation capacities predict positive and negative affective states of adolescence during the post-pandemic era. The finding from our study would add to current knowledge regarding the influence of the pandemic on adolescent's affective state, which will potentially inform establishing more sophisticated and effective mental health interventions among adolescence.

## Materials and methods

### Participants and procedures

A total of 436 high school students (40.80% male students), aged 14–19 years (mean age = 16.07 years and SD = 1.08), were included *via* a convenience sampling method in this cross-sectional survey from November 25 to December 3, 2021. This period was representative of the COVID-19 post-pandemic era when the epidemic was readily to break out in a small scale, especially in certain season, although widespread outbreaks of this infectious disease were effectively contained. In this case, life and work had not returned to normal as before this pandemic and social environment was full of impermanence. Many restrictions were placed by Chinese central and local governments to deal with this concern. The measures include traveling limits, social distancing, closure or lockdown of schools and workplaces, requirement of mask wearing in public places, and regular nucleic acid test. All these have been documented to be an etiology of negative emotions (Ge et al., 2021). The participants were enrolled from three schools in three provinces. All the respondents were informed beforehand regarding the purpose and process of the whole study. They were also told to participate in this study voluntarily and anonymously and have the opportunity to withdraw from this participation at any time. Participants that agreed to engage in fulfilled the questionnaires following the instruction of research assistants in the classroom. The inclusion criteria were as follows: (1) being high school students; (2) speaking and adequately understanding mandarin. Those with a history of major physical and psychiatric disorders were excluded from this study. This study was reviewed and approved by the Ethics Committee of Jinzhou Medical University. All procedures met the 1964 Helsinki Declaration and its later amendments. The participants and their legal guardian/next of kin provided their written informed consent to participate in this study. A total of 205 (47.00%), 43 (9.90%), and 188 (43.10%) participants were in their first, second, and third academic year, and 381 (87.40%) individuals reported their ethnicity as Han and 55 (12.60%) as an ethnic minority. The detailed demographic information of the study sample is shown in Table 1.

**Table 1:** Demographic characteristics of the study sample (*n* = 436).

			**PANAS-PA**				**PANAS-NA**			
**Variable name**	***N***	**%**	**Mean**	**SD**	***t*/*F***	***P***	**Mean**	**SD**	***t/F***	***P***
**Sex**										
Male	178	40.8	33.54	7.50	3.60	**<0.001**	27.40	7.87	1.04	0.30
Female	258	52.9	31.13	6.39			26.60	7.97		
**Grade**										
First	205	47.00	32.41	7.26	3.81	**0.02**	27.19	7.84	0.78	0.46
Second	43	9.90	29.35	7.08			25.53	7.93		
Third	188	43.10	32.41	6.48			26.95	8.03		
**Ethnicity**										
Han	381	87.4	32.02	7.16	−0.72	0.47	27.04	7.93	0.83	0.40
Ethnic minority	55	12.6	32.75	5.45			26.09	7.90		
**Only child**										
Yes	243	55.7	31.29	6.86	2.78	**0.01**	26.95	8.00	−0.07	0.95
No	193	44.3	33.15	6.97			26.90	7.86		
**Interpersonal relationship**										
Bad	18	4.12	26.56	7.62	31.16	**<0.001**	30.72	8.33	6.26	**<0.001**
Ordinary	142	32.57	29.25	6.05			28.28	7.27		
Good	276	63.3	33.95	6.68			25.98	8.08		
**Family economic status**										
Poor families	27	6.19	32.63	8.30	4.90	**0.01**	27.63	7.66	1.21	0.30
Middle-income families	396	90.8	31.89	6.78			26.77	7.84		
Rich families	13	3	37.92	7.40			30.08	10.66		
**Health status**										
Poor	16	3.67	28.19	6.79	23.87	**<0.001**	28.13	6.98	10.39	**<0.001**
Middle	141	32.34	29.32	6.04			29.28	7.54		
Good	279	63.99	33.75	6.89			25.66	7.91		
**Sleep condition**										
Poor	54	12.39	30.24	8.80	11.15	**<0.001**	30.31	8.61	6.86	**<0.001**
Middle	189	43.35	30.90	6.45			27.04	7.30		
Good	193	44.27	33.82	6.51			25.87	8.08		

Figures in bold represent being statistically significant at P < 0.05 level.

PANAS, Positive Affect and Negative Affect Scale; PA, positive affect; NA, negative affect; SD, standard deviation.

### Measures

#### PANAS

The Positive Affect and Negative Affect Scale (PANAS) consisted of 20 items in total, with 10 rating positive affect and another 10 negative affect (Watson et al., 1988). Each item was rated on a five-point Likert scale from 1 (very inconsistent) to 5 (very consistent), with the total score for each affect ranging from 10 to 50. Higher subscale score means more experience in positive or negative affect. The Chinese version of PANAS has been confirmed to be sufficiently reliable and valid (Man et al., 2004; Xue et al., 2021). In the present study, Cronbach's alpha coefficients for positive and negative affect subscales were 0.881 and 0.900, respectively.

#### ERQ

Ten-items ERQ was used to rate two distinct emotional regulation strategies: cognitive reappraisal (items 1, 3, 5, 7, 8, and 10) and expressive suppression (items 2, 4, 6, and 9) (Gross and John, 2003). Each item was scored on a seven-point Likert scale ranging from 1 (strongly disagree) to 7 (strongly agree). A higher total score for each subscale denotes a higher frequent usage of the corresponding emotion regulation strategy. The Chinese version of ERQ exhibited sound psychometric properties both in adults and adolescents (Liu et al., 2017). In this survey, the internal consistency Cronbach alpha coefficients were 0.884 (cognitive reappraisal) and 0.732 (expressive suppression), respectively.

#### DERS

The DERS consists of 36 items and is scored on a five-point Likert scale ranging from 1 (almost never) to 5 (almost always). This scale evaluated six dimensions of individual emotion regulation, including emotion perception, emotional understanding, goal-directed behavior stimulation, emotional impulse control, effective use of emotion regulation strategies, and emotional response acceptance (Gratz and Roemer, 2004; Zhang et al., 2022). Higher DERS sore in each dimension means worse ability at a certain aspect of emotion regulation. The Chinese version of DERS showed satisfactory factor structure, validity, and reliability (Li et al., 2018). Cronbach's alpha for these subscales ranged from 0.570 to 0.906 in this study.

#### ERSQ

The ERSQ was constructed based on the ACE model and ART to evaluate how individuals cope with negative emotions in the last week. This instrument contains 27 items to measure nine emotional regulation skills: awareness, clarity, sensation, understanding, compassionate self-support, modification, acceptance, tolerance, and readiness to confront. Each skill (subscale) is composed of three items rated on a five-point Likert scale from 0 (not at all) to 4 (almost always). According to research by Fujisato et al. (2017), these skills can be divided into two domains *Acceptance and Engagement (AE)* and *Awareness and Understanding (AU)*. In this study, Cronbach's alpha coefficients of the ERSQ for AE and AU were 0.886 and 0.906, respectively.

### Statistical analysis

All statistical analyses were conducted using the Statistical Packages for the Social Sciences (SPSS, version 22.0). The data were presented as mean, standard deviation (SD), and percentage (%). To primarily screen out the potential confounders that interfere with the association between emotion regulation strategies and affect state, Student's *t*-test and one-way analysis of variance (ANOVA) were used for comparison of difference in the levels of positive and negative affects among groups of varied demographic characteristics. Between-variable correlation was assessed using Pearson's correlation analysis. Multiple stepwise linear regression analysis using the forward selection method was used to evaluate factors associated with the outcome variables of affect state. Variables associated with the dependent variables with probability of *F* < 0.05 were entered into the multivariate analyses, and those larger than 0.1 were removed. Following repeated rounds of analysis through iteratively examining the statistical significance of each explanatory variable, a final model containing a set of explanatory variables that significantly influence outcome variables was constructed. Prior to performing linear regression analysis, we examined the data assumptions and confirmed they were met. Specifically, the linear relations between the outcome variables (positive and negative affects) and continuous predictors (the 10 emotion regulation strategies), independence of all data as examined by Durbin-Watson values, normality, homoscedasticity, and multicollinearity (absence) as measured by variance inflation factor (VIF) were all confirmed. A two-tailed *P* < 0.05 was deemed to be statistically significant.

## Results

### Comparison of affect state across demographic characteristics

Table 1 presents the scores of the PANAS-PA and PANAS-NA from diverse emotion regulation measures as per sex, grade, ethnicity, only-child status, interpersonal relationship, family economic status, health status, and sleep condition in our sample (*n* = 436). A significant difference in PANAS-PA and/or PANAS-NA scores was found with different levels/groups of these demographic characteristics except ethnicity. In summary, boys reported higher positive affect than girls. Students in grade two had the lowest positive affect. Compared with only-child Chinese high school students, the non-only-child individuals had a higher positive affect. Better interpersonal relationship was related to higher positive affect and lower negative affect. Those born in families reported highest scores both in positive and negative affects. Good health status and sleep quality were related to higher positive affect and lower negative affect.

### Pearson's correlation between emotion regulation strategies and PANAS-PA and PANAS-NA

The Pearson correlation coefficients between emotion regulation strategies and PANAS-PA and PANAS-NA are shown in Table 2. All the emotional regulation strategies were correlated with PANAS scores. Specifically, based on correlation coefficient values, relatively stronger correlation (|*r|* > 0.4) occurs in the following seven strategies: ERSQ-Acceptance and Engagement, ERSQ-Awareness and Understanding, ERQ-Cognitive reappraisal, DERS-Awareness, DERS-Acceptance, DERS-Impulse control, and DERS-Modification. The first four were related to positive affect and the other three negative affect.

**Table 2:** Correlation between emotion regulation strategies between PANAS-PA and PANAS-NA (*n* = 436).

	**PANAS-PA**	**PANAS-NA**
ERSQ-acceptance and engagement	0.529^**^	−0.147^**^
ERSQ-awareness and understanding	0.457^**^	−0.064
ERQ-cognitive reappraisal	0.523^**^	−0.210^**^
ERQ-expressive suppression	0.163^**^	0.124^**^
DERS-awareness	−0.420^**^	−0.100^*^
DERS-clarity	−0.143^**^	0.390^**^
DERS-acceptance	−0.004	0.523^**^
DERS-impulse control	−0.105^*^	0.489^**^
DERS-goals-directed engagement	−0.141^**^	0.353^**^
DERS-modification	−0.152^**^	0.502^**^

* P < 0.05.

** P < 0.01.

PANAS, Positive Affect and Negative Affect Scale; PA, positive affect; NA, negative affect; ERSQ, Emotion Regulation Skill Questionnaire; ERQ, Emotion Regulation Questionnaire; DERS, Difficulties in Emotion Regulation Scale; ERQ, Emotion Regulation Questionnaire; ERSQ, Emotion Regulation Skill Questionnaire; DERS, Difficulties in Emotion Regulation Scale.

### Multiple regression analysis of the correlation between diverse emotion regulation strategies and PANAS-PA and PANAS-NA

Multiple stepwise linear regression analysis results of the correlation between diverse emotion regulation strategies and PANAS-PA and PANAS-NA are shown in Table 3. After adjusting for diverse socio-demographic variables, the PANAS-PA score was significantly and positively correlated with ERQ-Cognitive reappraisal and ERSQ-Acceptance and Engagement, and negatively with DERS-Awareness. As for PANAS-NA, DERS-Acceptance and DERS-Modification were positively correlated.

**Table 3:** Multiple linear regression model for PNAS-PA and PNAS-NA (*n* = 436).

		***B***	**β**	***P***	**95.0% CI**		***R* ^2^**
PANAS-PA	Constant	18.91		0.00	14.23	23.59	0.46
	ERQ-cognitive reappraisal	0.37	0.32	0.00	0.26	0.47	
	ERSQ-acceptance and engagement	0.15	0.19	0.00	0.07	0.22	
	DERS-awareness	−0.26	−0.17	0.00	−0.40	−0.13	
	Interpersonal relationship (very bad^*^)
	Good	2.36	0.16	0.00	1.28	3.43	
	Grade (first^*^)
	Second	−2.52	−0.11	0.00	−4.15	−0.88	
	Health status (poor^**^)
	Good	1.36	0.10	0.01	0.35	2.38	
	Sex (male^**^)
	Female	−1.13	−0.08	0.03	−2.14	−0.12	
PANAS-NA	Constant	16.29		0.00	13.79	18.79	0.33
	DERS-acceptance	0.46	0.33	0.00	0.30	0.62	
	DERS-modification	0.27	0.23	0.00	0.13	0.40	
	Health status (poor^**^)
	Good	−1.75	−0.11	0.01	−3.10	−0.40	
	Interpersonal relationship (very bad^**^)
	Good	−1.50	−0.09	0.03	−2.83	−0.17	
	Grade (first^**^)
	Second	−2.09	−0.08	0.05	−4.16	−0.02	

* Reference group.

PANAS, Positive Affect and Negative Affect Scale; PA, positive affect; NA, negative affect; ERQ, Emotion Regulation Questionnaire; ERSQ, Emotion Regulation Skill Questionnaire; DERS, Difficulties in Emotion Regulation Scale.

## Discussion

The continuing duration of the COVID-19 pandemic, even during the post-pandemic era, has produced a negative chain reaction, including social distancing, public place/school closure, increased social media use, and elevated exposure to the electronic screen. All these unpleasant situations have triggered a global mental health crisis, not only provoking the increased incidence of psychiatric symptoms but also aggravating the preexisting mental health problems among adolescents (Golberstein et al., 2020; Khafif et al., 2022). In addition, amounting evidence also confirmed the worsening effect of the COVID-19 pandemic and related containment measures on emotion regulation skills (Golberstein et al., 2020). The present study is one of the few determining the influence of emotion regulation strategies on mood state among Chinese adolescents during the post-pandemic era of COVID-19. Through a comprehensive analysis using multiple emotion regulation models, we hope to provide clinicians valuable information regarding the determination of the most effective emotion regulation strategies to improve adolescents' mental health. In the present study, after adjusting for a range of socio-demographic variables, we in the end revealed that cognitive reappraisal, acceptance and engagement, awareness, and modification of emotions were significantly relevant to affect state of high school students.

The findings obtained from the present study revealed a positivity ratio of 1.19 (positivity: 32.11, negativity: 26.92) among our participants. Due to the absence of data regarding the pre-pandemic positivity ratio among Chinese adolescents, we are unable to make a proper comparison. However, the ratio value of 1.19 was far lower than the critical ratio of 2.9 that well discriminates flourishing from not flourishing mental health (Fredrickson and Losada, 2005; Diehl et al., 2011). After calculation, it was found that only 1.6% (7/436) adolescents in our present study reported a positivity ratio of ≥2.9, suggesting that the vast majority (98.4%) adolescents were living a not flourishing life in this special time. This low positive ratio is not surprising and is consistent with recent studies showing that the COVID-19 pandemic severely disrupted the wellbeing and mental health of youths (Wang et al., 2021; Bui et al., 2022; Petruzzelli et al., 2022).

In our sample, out of numerous emotion regulation strategies, four, including cognitive reappraisal, acceptance and engagement, awareness, and modification of emotions, were found to be independently associated with the positivity or negativity of our participants. These findings help address our initial concern regarding which emotion regulation strategies are potentially implicated in the modulation of affect state among Chinese high school students in the COVID-19 post-pandemic era full of unexpectedness and impermanence. Correspondingly, our findings emphasize the potential benefits of incorporating emotional regulation into mental health interventions *via* targeting the above four strategies toward Chinese adolescents living in times of the unprecedented and long-lasting post-pandemic era.

Based on our data, participants with higher level of cognitive reappraisal tended to be more positive in affect state. This implied that COVID-19 mentally had more impact on adolescents lower in reappraisal strategies. Cognitive reappraisal has been shown to be protective in both internalizing and externalizing psychiatric symptoms (Compas et al., 2017). As a primary target of cognitive behavioral and emotional regulation therapies, cognitive appraisal helps individuals experience more positive affect and less anxiety and depression (Brockman et al., 2017; Theurel and Gentaz, 2018). A recent study has revealed that cognitive reappraisal would decrease the negative impact of COVID-19 on anxiety, depressive symptoms, sleep disturbance, and proactive aggression among adolescents from the United States (Kuhlman et al., 2021). Notably, studies had shown that engagement in cognitive reappraisal emerges in the adolescence stage and thus is except to be quite malleable and easily developed (Silvers et al., 2012; Kuhlman et al., 2021). As such, cognitive appraisal training is projected to be able to confer lifelong psychological support for mental wellbeing of adolescents when they are confronted with challenging and long-lasting adversities, such as the COVID-19 pandemic and alike (Kuhlman et al., 2021).

According to Fujisato et al. (2017), acceptance and engagement indicates the ability to accept and behave without denying certain negative mood and reflected the ability of tolerance, modification, readiness to confront, and acceptance of emotion arousal during adversity. As per their results, these abilities were negatively associated with psychological distress and positively with life satisfaction (Fujisato et al., 2017). The beneficial effect of these emotion regulation capabilities was consistently seen in our sample population, the Chinese adolescents, as reflected by the prediction of acceptance and engagement on positive affect, as well as the prediction of acceptance difficulty on negative affect. In contrast to cognitive reappraisal, acceptance strategies do not require reframing emotional stimuli by subjective reassessment and recognition; alternatively, acceptance strategies emphasize the proactive acceptance of the negative emotion arousal and to let it be. Studies have shown that acceptance strategies are effective in lessening frustrating mood and depressive symptoms (Shiri et al., 2022; Zou et al., 2022). In addition, mounting randomized controlled trials or quasi-experimental studies have also confirmed that acceptance and commitment therapy (ACT) and mindfulness-based cognitive therapy (MBCT) are promising solutions to improving negative experience in adolescents, such as depression and anxiety (Pinhas-Hamiel and Hamiel, 2020; Musa et al., 2021; Shiri et al., 2022). As such, these two methods are expected to be feasible and effective for improving negative emotional experience in adolescents in the context of the ongoing COVID-19 pandemic and future comparable events.

In addition to the prediction of difficulty in acceptance in adolescents' affect state, we also presented evidence showing that awareness and modification difficulty were associated with decreased positive affective and increased negative affect, respectively. These findings were in line with a recent study conducted among Israeli adolescents which revealed that difficulties in emotional regulation significantly predicted mental health symptoms, including depression, anxiety, and emotional and behavioral problems (Hen et al., 2022).

Emotional awareness refers to paying attention to and being conscious about one's emotional response, and self/social-emotional regulation is one of the critical components of emotional intelligence (Shahid et al., 2018). Adequate emotional awareness is extremely significant for maintaining and promoting individuals' psychosomatic fitness (Kang and Shaver, 2004). Accumulating literature suggested that high emotional awareness facilitates affected individuals to escape negative mood (Salovey and Birnbaum, 1989). Also, emotion training seems more effective in those with high emotional awareness abilities (Zautra, 2003). Higher emotional awareness was frequently associated with a range of positive affect, such as better emotional self-regulation, elevated psychosomatic wellbeing, and better abilities to enjoy relationship (Lane and Smith, 2021). Based on these prior findings, alongside the prediction of emotional awareness on Chinese adolescents' affect state, it is proposed that targeting emotional awareness may be beneficial for affective intervention on the affected youth during the current special period. Based on these findings and existing scientific literature, mindfulness-based intervention might be a reliable strategy in improving mental health level of high-risk adolescents, as trait mindfulness results in unbiased identification of mood response, rendering them to conscientiously recognize, readily accept, and properly modify unpleasant emotion arousal, thereby improving emotion response process, and eventually decreasing mental distress (Froeliger et al., 2012; Ma and Fang, 2019). An encouraging empirical study also showed that 7-week mindfulness meditation can effectively disengage the subjects from negative emotional stimuli (Ortner et al., 2007). Whether mindfulness-based intervention improves adolescents' affect state warrants further exploration.

Previously, it was shown that high perceived lack of adaptive regulation strategies was uniquely associated with increased lifetime suicide attempts and recent suicidal ideation in patients with eating disorders (Rania et al., 2021). In our present study, difficulty in emotion modification was also shown to be significant in predicting increased negative emotion, suggesting the particular importance of access to functional emotional regulation strategies for adolescents experiencing COVID-19 pandemic-related stressors. As such, school-based emotional wellbeing program, such as Zippy's Friends (Clarke et al., 2014), should be tentatively applied in high school to increase students' emotional literacy and potential emotional and behavioral problems. In addition, other well-recognized emotional regulation strategy-oriented trainings that are confirmed to be applicable in adolescent population, such as regulatory emotional self-efficacy training (Wang et al., 2018) and dialectical behavior therapy (Turan and Akinci, 2022), may potentially benefit adolescents' mental health.

In addition to emotion strategies, affect state of our participants was also significantly influenced by multiple socio-demographic variables. These influencing factors included interpersonal relationship, health status, sex, and grade. For interpersonal relationship, our data showed that good interpersonal relationship predicted both increased positive affect and decreased negative affect. This is not hard to understand and in line with the previous finding that real-life interpersonal interaction may improve teenagers' emotion, sleep quality, and self-efficacy (Wang et al., 2020). Interaction with peers and parents plays a key role in teenagers' development. Furthermore, accumulating evidence has indicated that more positive interpersonal relationship with peers, teachers, or parents is in correlation with lower detrimental or negative emotions (La Greca and Harrison, 2005; Zimmer–Gembeck et al., 2007). As for health status, higher health level was associated with a higher positivity and lower negativity. In addition to subjective emotional content due to physically all right, one alternative important reason might be related to increased physical activity (PA) in individual with better health condition (Wang A. et al., 2022). A recent study has posited that lack of PA is significantly associated with psychiatric disorders among medical students during the COVID-19 pandemic (Ghali et al., 2022). In contrast, higher intensity of physical exercise increases the likelihood of subjective wellbeing in Chinese residents (Gan and Jiang, 2022). In our study, girls reported less positive affect than boys. This finding was also seen in a recent study, which proved that female adolescents presented a greater psychological vulnerability and lower level of positive mental health (Almeida et al., 2022). In another study, female adolescents also reported a higher level of depression, anxiety, and stress than male adolescents during home confinement due to the COVID-19 Omicron variant outbreak (Verma et al., 2011). The greater psychological vulnerability prevailing among female sex might be due to their more sensitive perception of stress and negative events in the context of the stressful and uncertainty-full COVID post-pandemic era (Verma et al., 2011). More focus should be put on adolescent girls to improve their affect state.

Interestingly, we found that grade two high school students simultaneously exhibited higher positive and negative affects. The high positivity of this population may be attributed to decreased academic and interpersonal stress when compared with their grade one and grade three counterparts. Grade one high school students are experiencing transition from junior high school to high school, and thus, adapting to the significantly increased learning difficulty as well as interaction with new peers and teachers would be a major challenge for them. Over time, grade two students have gradually developed relatively stable interpersonal relationship and been accustomed to the pace of learning and therefore have a higher positive affect. Although having the highest cognitive and learning capacities, the National College Entrance Examination (NCEE, Gaokao in Chinese pinyin) places a great academic and psychological pressure on grade three students. As for the utmost negativity in grade two students, one plausible explanation might be due to emotional instability at puberty, which warrants future investigation.

In addition, although positivity and/or negativity difference was also found significant among groups of other socio-demographic variables, including only-child status, family economic status, and sleep condition in univariate analysis, they did not enter the final regression model, suggesting untrue between-variable correlations due to potential confounding factors. Therefore, we are still unable to claim that the affect state of the high school students in this study is significantly and independently influenced by these socio-demographic differences.

Despite these important findings, several limitations should be taken into account. First, the cross-sectional nature of this study limits its causal inference. Second, although the sample was obtained from three different provinces across China, the representativeness of these subjects remains a concern due to the convenience sampling method. In addition, all the variables were measured based on self-reported manner and thus would bring about potential information bias.

Overall, our findings highlight the trans-diagnostic importance of emotional regulation strategies in the modulation of the mental health of the vulnerable youth population in China in the context of the COVID-19 crisis and comparable future conditions. In view of the continuous, multifaceted influence on adolescents' mental health of the ongoing pandemic, more effort should be made to leverage modifiable and targetable psychological resources, such as emotion regulation strategies, to benefit their coping abilities.

## Data availability statement

The raw data supporting the conclusions of this article will be made available by the authors, without undue reservation.

## Ethics statement

The studies involving human participants were reviewed and approved by the Ethics Committee of Jinzhou Medical University. Written informed consent to participate in this study was provided by the participants and their legal guardian/next of kin.

## Author contributions

All authors listed have made a substantial, direct, and intellectual contribution to the work and approved it for publication.
